# Supplementary material for: Extraction, identification and component analysis of exosome-like nanovesicles in Anoectochilus roxburghii (Wall.) Lindl
Source: PeerJ. 2025 Oct 13;13:e20182. doi: 10.7717/peerj.20182 (PMC12530199; doi:10.7717/peerj.20182)
Supplement: Supplemental Information 1 — (A) Rutin. (B) I soquercitrin. (C) Narcissoside. (D) Quercetin. (E) Kaempferol. (F) Isorhamnetin. [file peerj-13-20182-s001.docx]

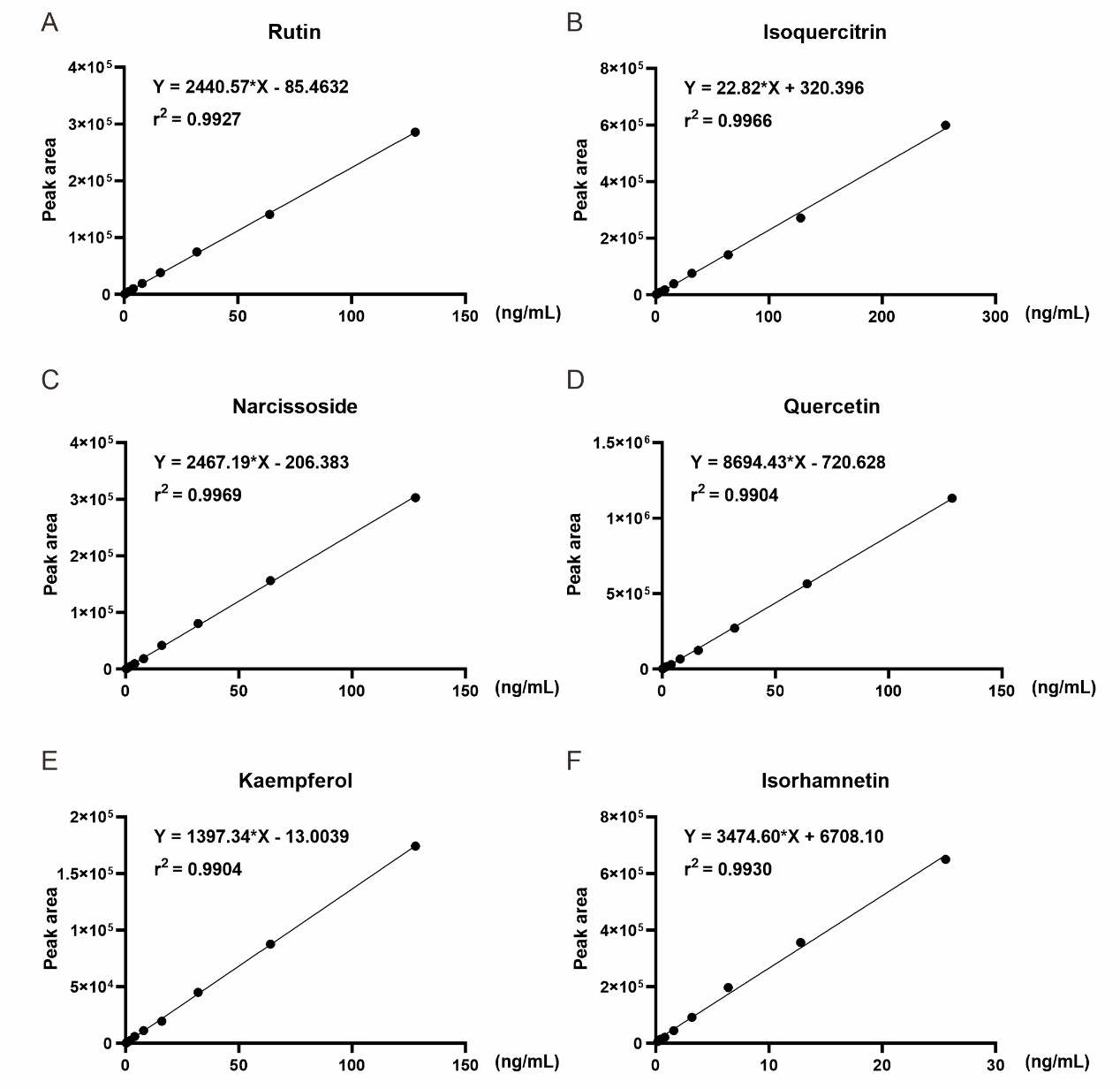


Figure S1. Standard curve and its corresponding standard equation of the six analytes. (A) Rutin. (B) Isoquercitrin. (C) Narcissoside. (D) Quercetin. (E) Kaempferol. (F) Isorhamnetin.
