# Supplementary material for: Extraction, identification and component analysis of exosome-like nanovesicles in Anoectochilus roxburghii (Wall.) Lindl
Source: PeerJ. 2025 Oct 13;13:e20182. doi: 10.7717/peerj.20182 (PMC12530199; doi:10.7717/peerj.20182)
Supplement: Supplemental Information 2 — All the data from Figures 1 to 4. [file peerj-13-20182-s002.zip › raw data/Figure 2B.pdf]

Size & Concentration Report

23 60×

Data File	20250430 23 60× 39.nfa	Population	Total

SN: FNAN30E20071514
Software: V2.0
Sample Pressure: 1.0Kpa

Laser: 5/40 mW 488
SS Decay: 10%
Threshold/sub: 63.6 9.2 1.9 1/0 0 0 0
Min Width: 0.3 ms

Total Size Infomation

Gating Range 30 - 150 nm

All Events	4837	
Gating Events	4128	
% of all	85.34	
Median	80.2	nm
Mean	85.7	nm
Std Dev.	20.2	nm

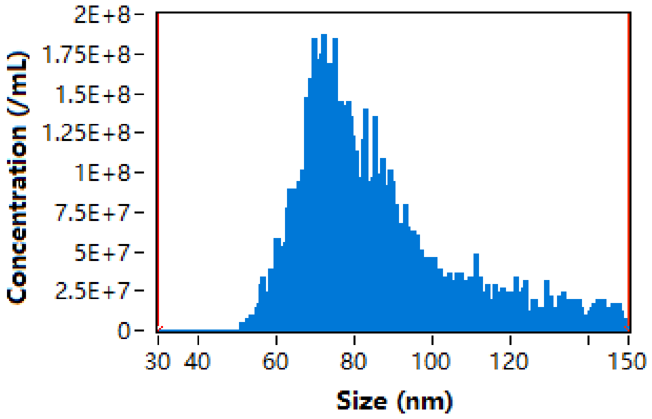

Total Concentration Infomation

	Particle Number	Dilution Factor
STD	5397	100
Blank	60	——
Sample	4897	60

STD Con.	2.16E+10	Particles/mL
Sample Flow Rate	24.99	nL/min
Sample Con.	1.16E+10	Particles/mL

Corrected Ratio: 4837/4837 100.0%

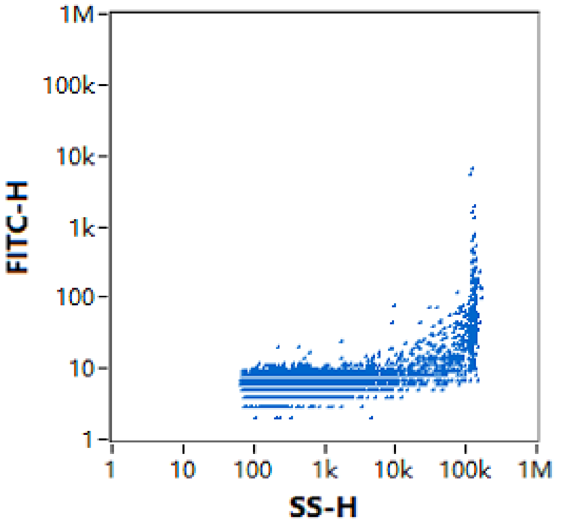

Report By :

2025/4/30 15:25
