# Supplementary figures and images for: Extraction, identification and component analysis of exosome-like nanovesicles in Anoectochilus roxburghii (Wall.) Lindl
Source: PeerJ. 2025 Oct 13;13:e20182. doi: 10.7717/peerj.20182 (PMC12530199; doi:10.7717/peerj.20182)

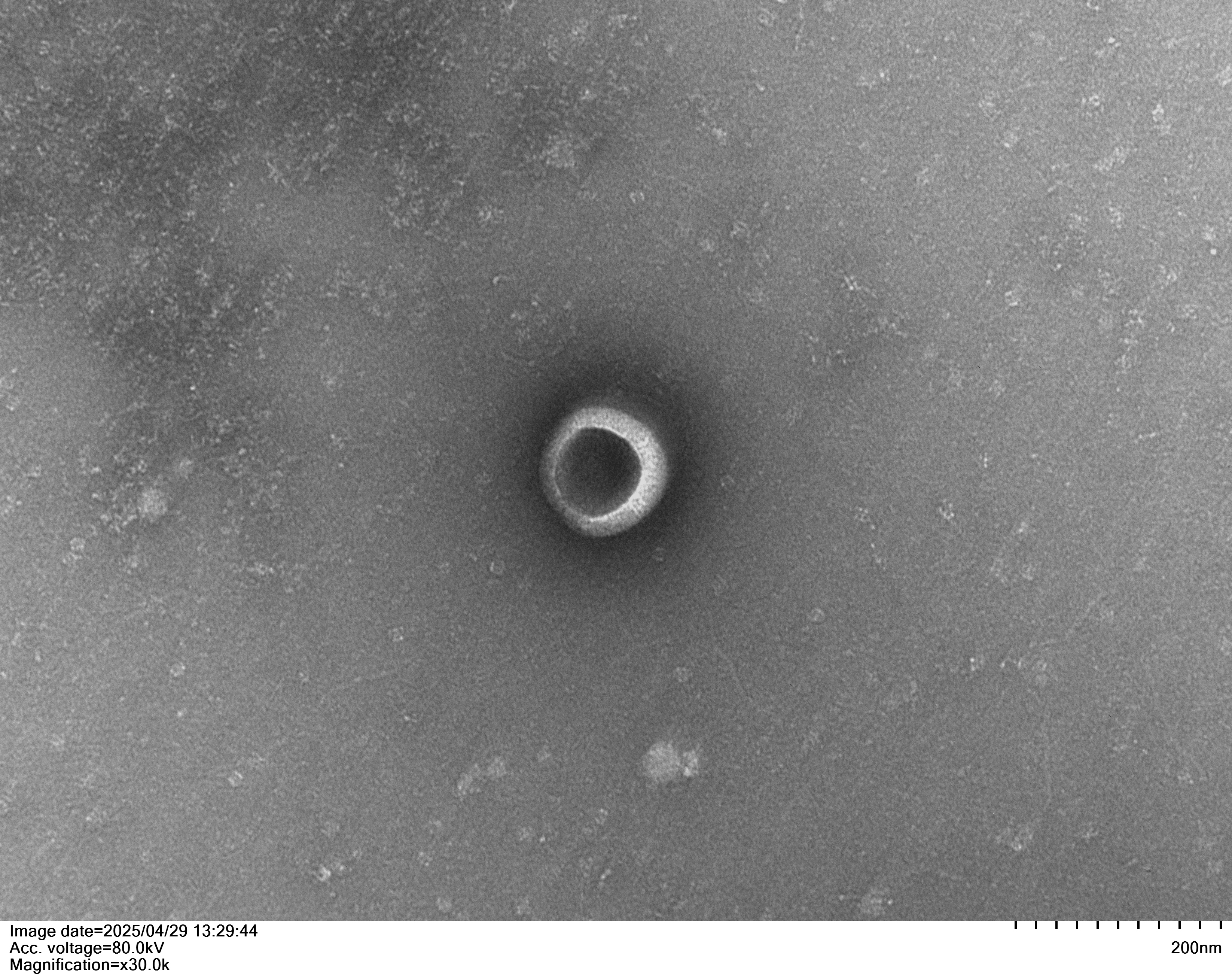

Supplement: Supplemental Information 2 — All the data from Figures 1 to 4. [file peerj-13-20182-s002.zip › raw data/Figure 2A.jpg]
